# Developing and Validating the Health Literacy Scale for Migrant Workers: Instrument Development and Validation Study

**DOI:** 10.2196/59293

**Published:** 2024-11-13

**Authors:** Soo Jin Kang, Hye-Kyung Oh, Hae-Ra Han

**Affiliations:** 1 Department of Nursing, Daegu University, Daegu, Republic of Korea; 2 School of Nursing, Johns Hopkins University, 525 N Wolfe St, Baltimore, MD, 21205-2110, United States, 1 4106142669, 1 4432870547

**Keywords:** transients and migrants, psychometrics, scale development, health literacy, validation study, Rasch model

## Abstract

**Background:**

Research concerning health literacy among migrant workers in South Korea has been limited, especially given the lack of validated instruments and the lack of focus on the cultural diversity of migrant workers.

**Objective:**

This study aimed to develop and validate a health literacy scale for unskilled migrant workers (HLS-MW) in South Korea.

**Methods:**

We first generated a pool of potential items based on a literature review and in-depth interviews with 23 migrant workers. Subsequently, we reviewed empirical referents from the first step to select relevant medical terminologies and passages, ultimately choosing 709 words. The study team initially generated 35 items with 709 health-related terms through empirical referent reviews. After content validity testing by an expert panel, 28 items comprising 89 terms on the 2 subscales of prose and documents were selected for psychometric testing. Overall, 402 unskilled migrant workers in South Korea completed a web-based survey between August and September 2021, with 334 responses included in the final analysis. We used multiple analytic approaches, including exploratory factor analysis, Rasch analysis (item response theory), and descriptive analysis, to examine the new scale’s validity and reliability.

**Results:**

The final sample primarily included young male workers from South Asian countries. The HLS-MW yielded 2 factors: prose and documents. The item difficulty scores ranged from −1.36 to 2.56. The scale was reduced to 13 items (10 prose and 3 document items), with the final version exhibiting good internal reliability (Kuder-Richardson index=0.88; intraclass correlation coefficient=0.94, 95% CI 0.93‐0.95) and test-retest reliability (*r*=0.74, 95% CI 0.57‐0.92). HLS-MW scores differed significantly by Korean language proficiency (*F*_2,331_=3.54, *P*=.004).

**Conclusions:**

The HLS-MW is a reliable and valid measure to assess health literacy among migrant workers in South Korea. Further studies are needed to test the psychometric properties of the HLS-MW in diverse migrant groups in South Korea while also establishing cutoffs to help identify those in need of health literacy support.

## Introduction

Globally, the number of migrant workers has increased over the past 2 decades, with approximately 61% of the estimated 169 million migrant workers working in high-income countries [1]. Since 1995, when the South Korean government introduced the Employment Permit System (EPS) for migrant workers [2], South Korea has transformed from a migrant-sending country into a migrant-receiving one [3,4]. In 2023, there were about 229,476 unskilled migrant workers in South Korea, representing the largest proportion of migrant workers in the country, excluding overseas Koreans (approximately 403,404) [5].

Most low-skilled migrant workers in South Korea are engaged in low-wage occupations [6] and tend to experience higher rates of adverse occupational effects, such as workplace injuries and poor working conditions (eg, higher burnout levels, sick leave, bullying in the workplace), compared to those engaged in skilled work [7,8]. Given the employment qualification criteria [2], low-skilled migrant workers in South Korea often lack Korean language proficiency, limiting their ability to communicate with health providers and acquire necessary health-related information [9,10]. COVID-19 exacerbated the inequities in the occupational safety and health outcomes of migrant workers [11-13]. In fact, the health status of low-skilled migrant workers was a serious public health issue during this time [14].

Social determinants of health are the social and economic factors that influence health outcomes and have been recognized by the World Health Organization (WHO) Commission on the Social Determinants of Health as having a notable role in individuals’ health status [15]. Health literacy is defined as “one’s ability to access, understand, and process health information and services needed to make decisions related to health,” and represents an important social determinant of health [16]. Specifically, migrant workers with inadequate language skills experience considerable barriers to accessing health care services, leading to multiple adverse health consequences, including poor health status and low quality of life [17-20].

Limited research has examined health literacy and health outcomes among migrant workers in South Korea due, in part, to the lack of a standardized scale to assess health literacy in this population [21]. Existing studies that have measured health literacy among migrant workers in South Korea did not use validated instruments [18,22]. Further, most migrant workers in South Korea are from South Asian countries (eg, Bangladesh and Cambodia) with different health systems, languages, and cultural backgrounds [23]. However, research has primarily focused on Korean-Chinese migrant workers who share similar cultural traditions and language roots [20]. To compensate for this research gap, this study aimed to develop and validate a health literacy scale for unskilled migrant workers (HLS-MW) in South Korea. This scale could allow health providers and public health practitioners to collect essential information regarding the needs and health literacy status of migrant workers in South Korea.

## Methods

### Study Design

This instrument development and descriptive study involved the development of an HLS-MW and the evaluation of the validity and reliability. The study included 2 phases—instrument development and instrument validation.

### Instrument Development

We developed the HLS-MW based on Baker’s health literacy framework [24]. According to this framework, health literacy involves one’s skills in different dimensions, such as health-related print (ie, documents, prose, and numeracy) and oral literacy. Within our HLS-MW scale, we focused on health-related print literacy—documents (ie, understanding forms and tables) and prose (ie, understanding health-related terms presented in sentences). The scale did not include numeracy as a separate dimension. Numeracy refers to the application of arithmetic operations and the understating of quantitative information [24]. We considered numeracy as a part of the “document” dimension in that it operationalizes one’s ability to understand written “quantitative” information. Given that the HLS-MW was not specific to a certain disease (eg, diabetes) and was intended to be used to assess general health literacy in migrant workers, we decided that there was a limited need to address specific arithmetic skills.

We reviewed quotes and themes from a qualitative study involving migrant workers to identify relevant venues in which migrant workers experience and encounter health literacy tasks in South Korea [20]. We also examined other empirical referents, such as health education materials, existing health literacy instruments [25-27], textbooks on the EPS—Test of Proficiency in Korean (EPS-TOPIK), resources for immigrants provided by the National Institute of Korean Languages, and relevant studies on immigrants’ health care in South Korea [20,28,29]. We extracted 20 passages and 709 health-related terms for migrant workers from these sources.

Document literacy included medical forms commonly used when visiting a clinic, such as appointment instructions for outpatient and lab visits. We used existing health literacy scales written in Korean [26,27] to modify the items that were salient to migrant workers, such as adapting items into plain language use based on word difficulty [30]. For prose literacy, the initial pool of 709 terms was categorized based on their characteristics (ie, body-related terms, disease- and symptom-related terms, treatment-related terms, and healthy life and miscellaneous) and difficulty (ie, beginner, intermediate, and advanced) to develop 35 items [30].

To examine the content validity of the 229 terms, a review was carried out by a panel comprising 7 health professionals, including 3 nurses, a social worker, a pharmacist, and 2 doctors, with at least 3 years of experience as volunteers at a free clinic for migrant workers.

**Figure 1.**
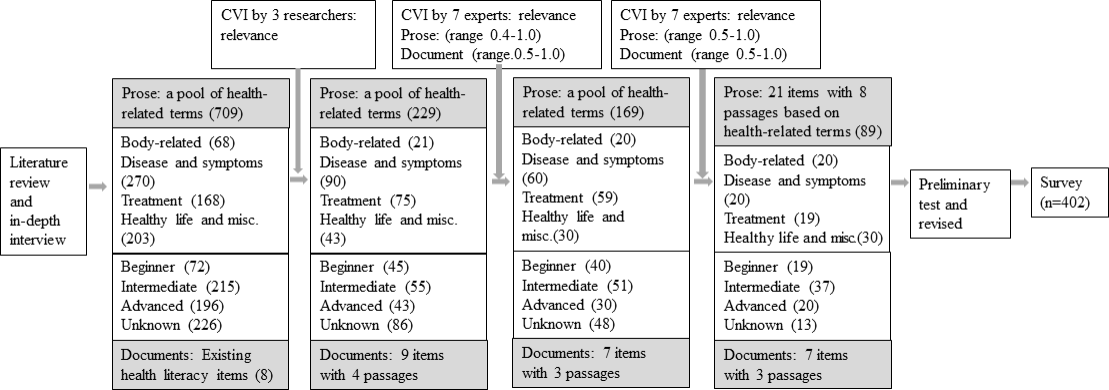
Item generation and testing process. CVI: content validity index.

Using a 4-point scale, they were asked to rate each document and prose item for its appropriateness (1=*not appropriate* to 4=*very appropriate*) and importance (1=*not important* to 4=*very important*). The content validity index (CVI) was 0.76 (range 0.43‐1), and any item with a CVI <0.7 was modified or removed [31], resulting in a final set of 28 items (7 document literacy items and 21 prose items). These items comprised 89 health-related terms embedded across 11 passages that addressed checking in at the clinic desk or doctor’s office and instructions for the next appointment. Subsequently, we conducted a field test with 10 migrant workers to confirm this initial version’s readability, understandability, and cultural relevance before proceeding with a validation process for psychometric testing. The process of the HLS-MW development is presented in Figure 1.

### Instrument Validation

We used web-based sampling to recruit participants for the validation study. First, in collaboration with the Support Center for Foreign Workers in Daegu, South Korea, we identified 6 migrant community leaders who could speak fluent Korean, representing 6 South Asian countries—Indonesia, Vietnam, Nepal, Cambodia, Pakistan, and the Philippines. Subsequently, we held a 1-hour briefing session with these 6 community leaders at the Daegu Center, where we explained the study’s purpose and how we planned to recruit study participants. The 6 leaders were granted access to a website written in their own language and asked to check the survey’s logic and any errors. If no errors were identified, each leader was provided with a link to the study survey written in their own language to recruit their peers in South Korea by sharing the link through mobile phone texts or social media between August 19 and September 2, 2021. Upon clicking the study link, potential participants were presented with a brief eligibility screening questionnaire, and after verifying their eligibility, eligible migrant workers were directed to a consent script followed by the survey. In addition to the HLS-MW, the web-based survey included sociodemographic (eg, nationality, age, sex, education, job, length of stay in South Korea, type of visa, health insurance, and Korean language proficiency) and medical status questions (eg, health problems and use of medical services in Korea). Participants’ Korean language ability was self-rated on a 4-point scale (0=*not proficient* to 3=*proficient*) across four areas (ie, reading, writing, listening, and speaking).

### Participants

A total of 402 migrant workers residing in South Korea responded to the web-based survey. Inclusion criteria encompassed (1) being previously or currently employed through the EPS for migrant workers, (2) having lived in South Korea for at least 6 months, (3) providing informed consent to participate in this study using their phone number, and (4) being able to read Korean. Potential participants were excluded if they were (1) international students, (2) earning an income from temporary economic activities on a tourist visa, or (3) Koreans living abroad (H-2 visa holders), since their Korean language proficiency, status of residence, and employment standard and procedures differed from those of unskilled migrant workers.

### Test-Retest Reliability

Of the 40 workers who completed the study survey and were asked to complete the HLS-MW again 2 weeks later, 23 complied (response rate of 57.5%).

### Statistical Analysis

We undertook a preprocessing step to identify and remove incomplete or invalid data, excluding 68 out of 402 surveys that were completed in less than 3 minutes. The 3-minute cutoff was based on the time it took for 5 native Korean workers with similar ages, education levels, and job categories to complete the study survey. Consequently, the final analytic sample included 334 complete surveys. The HLS-MW comprised 35 true-or-false items. An incorrect answer for each item was assigned a score of 0 and a correct answer was assigned a score of 1.

A descriptive analysis was conducted to summarize participants’ demographics and health literacy scores. Exploratory factor analysis (EFA) was performed via the Mplus 8.2 program (Muthén & Muthén). Weighted least square mean and variance adjusted (WLSMV) was determined using M-plus. The WLSMV approach is generally applied for dichotomous variables [32]. To determine the number of factors that should be retained in EFA [33], a scree plot of the eigenvalues of the tetrachoric correlation matrix was graphed. This study assessed the model fit by using root mean square error of approximation (RMSEA), the Tucker-Lewis index (TLI), the comparative fit index (CFI), and standardized root mean square residual (SRMR). The overall fit of the structural model was good, with the CFI and TLI above the recommended value of 0.9 [34], RMSEA less than 0.08 [35], and SRMR less than 0.08 [34]. Items with factor loadings of less than 0.4 were deleted [36].

The Rasch model, known as the 1-parameter logistic model under the item response theory (IRT) model, was applied to assess the adequacy of items in the measurement instrument; that is, all test items were intended to measure a single latent trait or construct [37]. Within the Rasch model, trait ability, item difficulty, and item-fit indices can be used to examine the psychometric properties of an instrument within a given population. In this view, a response to an item is caused by the (1) latent trait level of the subject (ability) and (2) agreement tendency of the item (difficulty). Conversely, item-fit indices are used to indicate the degree to which individual items define the unidimensionality of a construct.


$$P\left(x=1\right)=\frac{exp\left(B_{n}-D_{i}\right)}{1+exp\left(B_{n}-D_{i}\right)}$$

where *P*(*x*=1) is the probability of an endorsed response (a yes response to an item), *B_n_* is the trait (or ability) parameter of person *n*, and *D_i_* is the difficulty of endorsing item *i*. When *B_n_>D_i_, B_n_=D_i_*, and *B_n_<D_i_*, the chances of a yes response are greater than 50%, equal to 50%, and less than 50%, respectively [38].

There was an examination of the unidimensionality of items within the 2 dimensions of the HLS-MW (the assumption that items measure a single latent trait) and those items’ local independence (the assumption that responses to items are unrelated). To provide evidence in support of scale unidimensionality, item-fit indices statistics were computed using the jMetrik 4.0 program (Informer Technologies). Item-fit indices indicate the degree to which individual items define the unidimensionality of a construct [39]. Item fit was evaluated using outfit mean-square fit statistics (MNSQ) instead of standardized fit statistics (ZSTD) and infit MNSQ. MNSQ values of 1.0 are ideal, while values greater than 1.3 or less than 0.7 may indicate unsatisfactory model-data fit [40]. In the case of overfitting and underfitting items and the standardized transformation of the mean-square to approximate a *t* statistic (ZSTD), values greater than ±1.96 were considered potentially misfitting [37]. We examined the residual correlation matrix to check for values <0.2, indicative of potential local dependence [41].

We evaluated item difficulty and discrimination using classical test theory (CTT). Item difficulty levels range from 0 to 1, with higher values representing easier questions (ie, <0.25: difficult, 0.25-0.75: moderately difficult, and >0.75: easy) [42]. By contrast, a discrimination index closer to 1 indicates better discrimination (ie, >0.2: no item discrimination, 0.2-0.29: little discrimination [item revision warranted], 0.3-0.39: moderate discrimination [little or no revision required], >0.4: satisfactory discrimination).

Based on Everitt’s recommendations [43] for sample size in factor analysis (participant to variable ratio of 10:1), a minimum sample size of 280 was needed for the study. Additionally, with the Rasch model’s application, at least 200 respondents were needed for successful estimation [38]. Therefore, a sample size of 334 was considered adequate. Final items were selected based on item difficulty, model composition, goodness-of-fit, factor loadings, content validity, and item redundancy. In particular, the number of items with similar difficulty levels was reduced by discarding some redundant items [44].

Finally, test-retest reliability, item-total correlations, internal consistency, and intraclass correlation coefficients (ICCs) were examined to address the reliability of the HLS-MW. Specifically, item-total correlations greater than 0.3 were considered adequate [45], and the Kuder-Richardson index was used to estimate internal consistency reliability.

For construct validity testing, we hypothesized that health literacy scores would be associated with sex, education, and Korean language proficiency. We performed 2-tailed *t* tests and ANOVAs to explore the relationships between these factors and health literacy. Based on existing research [22], we hypothesized that men with higher education and those with higher Korean language proficiency would have significantly higher health literacy scores using the HLS-MW.

### Ethical Considerations

This study was approved by the institutional review board of Daegu University (IRB no. 1040621‐202101HR-005) before data collection. All participants were informed of the purpose of the study. Written informed consent was obtained from every participant before beginning data collection.

## Results

### Validation Sample Characteristics

Table 1 shows the sample characteristics (N=334). The sample comprised mostly males (n=277, 82.9%) in their 30s (mean age 31.89, SD 5.55 years). The majority of participants were from Indonesia (99/334, 29.6%) and Vietnam (93/334, 27.8%), followed by the Philippines (50/334, 15%), Nepal (41/334, 12.3%), Cambodia (35/334, 10.5%), and Pakistan (14/334, 4.2%). In terms of length of residence, most had resided in Korea for 5‐10 years (122/334, 36.5%) or less than 5 (183/334, 54.8%) years. More than half (170/334, 50.9%) of the sample had completed high school. Most participants (279/334, 83.5%) did not have current health problems and had health insurance (303/334, 90.7%). Regarding their Korean language proficiency, only 9.6% (32/334) were fluent, whereas 38.3% (128/334) had difficulty with Korean, and 52.1% (174/334) had average proficiency. Out of 28 items, 11 (3.3%) answered all items incorrectly; no one answered all items correctly, and 4 (1.2%) people answered 27 questions correctly (Multimedia Appendix 1).

**Table 1.** General characteristics of participants (N=334).

Characteristics		Values
**Sex, n (%)**		
	Male	277 (82.9)
	Female	57 (17.1)
**Age, years (range 22‐50), n (%)**		
	20‐29	125 (37.4)
	30‐39	178 (53.3)
	>40	31 (9.3)
	Mean (SD)	31.89 (5.55)
**Nationality, n (%)**		
	Indonesia	99 (29.6)
	Vietnam	93 (27.8)
	The Philippines	50 (15)
	Nepal	41 (12.3)
	Cambodia	35 (10.5)
	Pakistan	14 (4.2)
	Other	2 (0.6)
**Job, n (%)**		
	Manufacturing	302 (90.4)
	Agriculture	20 (6)
	Other	12 (3.6)
**Length of stay in Korea, years (range 0.6‐92.2)** ^**a**^ **, n (%)**		
	<5	183 (55.6)
	5‐10	122 (36.9)
	>10	25 (7.5)
**Education** ^**a**^ **, n (%)**		
	Middle school	43 (12.9)
	High school	170 (51.4)
	Some college (2‐3 years)	72 (21.8)
	University or higher	46 (13.9)
**Visa status, n (%)**		
	Documented	324 (97)
	Undocumented	10 (3)
**Health insurance, n (%)**		
	Yes	303 (90.7)
	No	31 (9.3)
**Current health problems, n (%)**		
	Yes	55 (16.5)
	No	279 (83.5)
**Korean proficiency (range 4‐20), n (%)**		
	Not fluent	128 (38.3)
	Average	174 (52.1)
	Fluent	32 (9.6)
**Medical service use in the past year, n (%)**		
	Yes	129 (38.6)
	No	205 (61.3)

a Missing data included.

### EFA Examination

An EFA was conducted to examine the factor structure and dimensionality of the data, with the findings showing that the unidimensionality assumption was not violated. The EFA revealed three different models (1-, 2-, and 3-factor). All models had a good fit with no significant differences (Table 2). However, the 1-factor and 3-factor models did not match the conceptual structure of the HLS-MW. In the 2-factor model, most items were significantly loaded on each factor (ie, factor loading>0.4) except for items 24 and 28 (Table 2). Nevertheless, the EFA fit indices overall supported the 2-factor structure of the HLS-MW, with the first factor accounting for 35.4% of the total variance (Multimedia Appendix 2).

**Table 2.** Exploratory factor analysis with factor loadings.

Item	Variables	1-factor model^a^	2-factor model^b^		3-factor model^c^		
		Factor 1	Factor 1	Factor 2	Factor 1	Factor 2	Factor 3
1	At the reception desk—check-in	0.54	0.69	−0.17	0.54	0.25	−0.04
2	Blood pressure	0.53	0.70	−0.20	0.43	0.45	0.01
3	Temperature	0.69	0.85	−0.19	0.62	0.39	0.00
4	At the doctor’s office 1—cough	0.60	0.74	−0.17	0.53	0.36	0.00
5	Breathe	0.74	0.89	−0.17	0.79	0.19	−0.07
6	Antibiotics	0.75	0.70	0.07	0.42	0.42	0.30
7	Symptoms	0.79	0.73	0.09	0.57	0.22	0.23
8	At the doctor’s office 2—ointment	0.78	0.76	0.05	0.66	0.14	0.13
9	Painkiller	0.79	0.88	0.09	0.91	−0.02	−0.10
10	Physical therapy	0.69	0.60	0.13	0.56	0.03	0.17
11	At the doctor’s office 3—allergy	0.68	0.84	−0.18	0.77	0.15	−0.11
12	Pain relief patches	0.72	0.70	0.04	0.71	−0.03	0.04
13	Water	0.85	0.82	0.05	0.79	0.02	0.08
14	Assessment instruction 1—blood sampling room	0.69	0.64	0.07	0.65	−0.03	0.07
15	Prescription	0.84	0.74	0.14	0.69	0.04	0.19
16	Assessment instruction 2—fast	0.78	0.62	0.22	0.60	−0.02	0.24
17	Chaperon (guardian)	0.74	0.63	0.16	0.73	−0.21	0.09
18	Outpatient	0.71	0.61	0.13	0.68	−0.15	0.08
19	Health management 1—sleep	0.79	0.80	0.00	0.89	−0.15	−0.06
20	Health management 2—health examination	0.87	0.62	0.33	0.69	−0.23	0.29
21	Smoking	0.78	0.78	0.01	0.84	−0.10	−0.02
22	Appointment instruction (day)	0.73	−0.02	0.87	0.01	−0.23	0.86
23	Appointment instruction (place)	0.69	0.03	0.79	0.00	−0.12	0.81
24	Appointment instruction (time)	−0.27	0.28	−0.67	0.36	0.00	−0.74
25	Outpatient schedule (department)	0.87	0.30	0.67	0.18	0.09	0.75
26	Outpatient schedule (day)	0.87	0.09	0.90	−0.02	0.03	0.99
27	Nutrition facts	0.65	0.15	0.62	0.08	−0.01	0.67
28	Sodium	0.41	0.36	0.08	0.43	−0.13	0.02

a Model fit: *χ*2_350_=737.51; *P*<.001; root mean square error of approximation=0.05; standardized root mean square residual=0.09; comparative fit index=0.95; Tucker-Lewis index=0.95.

b Model fit: *χ*2_323_=472.41; *P*<.001; root mean square error of approximation=0.03; standardized root mean square residual=0.06; comparative fit index=0.98; Tucker-Lewis index=0.98.

c Model fit: *χ*2_297_=389.69; *P*<.001; root mean square error of approximation=0.03; standardized root mean square residual=0.05; comparative fit index=0.98; Tucker-Lewis index=0.98.

### Evaluation of IRT Assumption

The unidimensional assumption should exceed 20% of the accounted variance—as per Reckase’s rule [46]—in the first factor, and the scree plot of the eigenvalues should be greater than 1.0 [34]. The scree plot appeared to show one dominant factor; hence, the scale was deemed sufficiently unidimensional (Multimedia Appendix 2). The absolute value of the correlation coefficient between the residuals of the item was less than 0.2; therefore, local independency was not violated [41].

### Item Analysis and Item Selection

Table 3 shows item difficulty, item discrimination, and item fit based on CTT and the Rasch model. According to CTT, item difficulty in the prose literacy section ranged from 0.33 to 0.7, and item discrimination ranged from 0.39 to 0.69. Among the 21 items, most showed moderate difficulty with acceptable discrimination. In the document literacy section, item difficulty ranged from 0.12 to 0.63, and item discrimination ranged between −0.15 and 0.64; item 24 had a negative value (−0.15), indicating low validity. Additional results of the theta (IRT ability scores) and IRT test information function are presented in Multimedia Appendices 3 and 4.

According to the Rasch model for item fit, 17 prose items and 5 document items showed a satisfactory fit, while an unsatisfactory fit was found in 6 items (items 1, 2, 4, 18, 24, 28). However, item 2 was retained because the degree of deviation from the fit indices and ZSTD value was marginal, increasing the content validity. Items 7, 13, and 21 were removed as these items had difficulty values similar to those of item 3. Additionally, item 28 was removed due to its difficulty value for migrant workers.

To address item redundancy, items 22, 26, 6, and 10 (−1.19, −0.89, −0.05, −0.05, respectively) and items 12, 11, and 14 (0.15, 0.31, and 0.35, respectively) were eliminated due to similar difficulty levels. Considering the item difficulty, composition, goodness-of-fit, and factor loading, the final HLS-MW consisted of 13 items (10 items addressing prose literacy and 3 items addressing document literacy).

**Table 3.** Item characteristics, model fits, and final items.

Item		Classical test theory		Rasch analysis			Reliability (item-to-total correlation)	Selected final item
		Item difficulty	Item discrimination	Item difficulty	Infit (MNSQ^a^)	Outfit (MNSQ)		
**Prose literacy**								
	1	0.52	0.43	−0.11	1.28	1.58	0.48	
	2	0.34	0.39	1.15	1.32	1.52	0.44	✓
	3	0.56	0.53	−0.39	1.04	0.93	0.58	✓
	4	0.56	0.46	−0.39	1.18	1.37	0.51	
	5	0.47	0.59	0.21	0.93	0.79	0.63	✓
	6	0.51	0.59	−0.05	0.99	1.04	0.63	
	7	0.55	0.63	−0.33	0.91	0.77	0.67	
	8	0.63	0.62	−0.82	0.91	0.89	0.65	✓
	9	0.48	0.63	0.17	0.88	0.87	0.67	✓
	10	0.51	0.55	−0.05	1.08	1.17	0.59	
	11	0.46	0.54	0.31	1.04	0.98	0.59	
	12	0.48	0.56	0.15	1.04	1.01	0.61	
	13	0.55	0.68	−0.31	0.79	0.81	0.72	
	14	0.45	0.54	0.35	1.08	1.05	0.59	
	15	0.54	0.69	−0.27	0.81	0.66	0.72	✓
	16	0.58	0.61	−0.51	0.96	0.88	0.65	✓
	17	0.45	0.58	0.37	0.99	1.09	0.62	✓
	18	0.35	0.53	1.06	1.03	1.40	0.58	
	19	0.33	0.58	1.19	0.89	1.02	0.62	✓
	20	0.70	0.65	−1.36	0.86	0.61	0.68	✓
	21	0.55	0.61	−0.31	0.92	0.81	0.65	
**Document literacy**								
	22	0.60	0.52	−1.19	0.86	0.68	0.56	
	23	0.60	0.50	−1.17	0.93	0.79	0.54	✓
	24	0.12	−0.15	2.56	1.62	9.05	−0.11	
	25	0.63	0.64	−1.36	0.80	0.57	0.67	✓
	26	0.57	0.62	−0.89	0.61	0.49	0.66	
	27	0.48	0.49	−0.21	0.92	0.83	0.53	✓
	28	0.15	0.25	2.26	0.95	1.90	0.30	

a MNSQ: mean-square fit statistics.

### HLS-MW Scores by Key Demographic Characteristics

Table 4 shows a comparison of HLS-MW scores according to key demographic characteristics. We found that age was significantly associated with health literacy scores among migrant workers (*F*_2,331_=3.11, *P*=.04). Specifically, those aged 30‐39 years had a significantly higher health literacy score (mean 7.03, SD 4.18) compared to those aged 20‐29 (mean 6.84, SD 3.66) years and those aged above 40 (mean 5.09, SD 4.26) years. Participants who worked in manufacturing had significantly higher HLS-MW scores than those who worked in agriculture groups (*t*_2,331_=3.64, *P*=.02). Additionally, migrant workers residing in South Korea for 5‐10 years had the highest HLS-MW scores, followed by those with shorter (<5 years) and longer (>10 years) years of residence (*F*_2,327_=3.15, *P*=.03). HLS-MW scores were high in the order of Korean language proficiency (*F*_2,331_=3.54, *P*=.004). Finally, participants who completed high school had the highest HLS-MW scores (mean 7.04, SD 4.02), followed by those who completed college or higher (mean 6.79, SD 4.09) and middle school (mean 6.02, SD 3.89) levels of education. However, these differences in health literacy scores by educational level were not statistically significant.

**Table 4.** Descriptions in the health literacy scale for unskilled migrant workers (HLS-MW) scores according to general characteristics.

Characteristics		n	Score, mean (SD)	*F/t* (*P* value)
**Sex**				−0.12 (.91)
	Male	277	6.77 (4.05)	
	Female	57	6.84 (3.99)	
**Age (years)**				3.11 (.04)
	20‐29	125	6.84 (3.66)	
	30‐39	178	7.03 (4.18)	
	≥40	31	5.09 (4.26)	
**Nationality**				1.90 (.08)
	Indonesia	99	7.58 (3.81)	
	Vietnam	93	7.22 (4.34)	
	Philippines	50	6.00 (3.41)	
	Nepal	41	6.12 (4.54)	
	Cambodia	35	5.69 (3.96)	
	Pakistan	14	6.00 (3.42)	
	Other	2	5.50 (2.12)	
**Job**				3.64 (.02)
	Manufacturing	302	6.95 (4.03)	
	Agriculture	20	4.50 (3.44)	
	Others	12	6.25 (4.09)	
**Length of residence (years)**				3.15 (.03)
	<5	183	6.46 (3.67)	
	5‐10	122	7.43 (4.34)	
	≥10	25	5.64 (4.70)	
**Education^a^**				1.10 (.33)
	Middle school	43	6.02 (3.89)	
	High school	170	7.04 (4.02)	
	Some college or higher	118	6.79 (4.09)	
**Type of visa**				−0.17 (.86)
	Documented	324	6.78 (4.02)	
	Undocumented Status	10	7.00 (4.78)	
**Health insurance**				1.04 (.29)
	Yes	303	6.86 (3.99)	
	No	31	6.06 (4.48)	
**Current health problems**				1.42 (.16)
	Yes	55	7.49 (4.29)	
	No	279	6.65 (3.98)	
**Korean language proficiency**				3.54 (.004)
	Not proficient	128	6.22 (3.93)	
	Average	174	6.93 (4.07)	
	Proficient	32	8.25 (3.89)	

a Missing data included.

### Reliability

The HLS-MW exhibited good internal consistency. The Cronbach α coefficient was 0.88 for the overall scale, 0.86 for the prose literacy section, and 0.67 for the document section. The ICC was 0.94 (95% CI 0.93‐0.95) and the test-retest reliability was *r*=0.74 (95% CI 0.57‐0.92).

## Discussion

### Principal Findings

This study is one of the first to systematically assess health literacy among unskilled migrant workers. We developed a theory-based health literacy scale—HLS-MW—and validated it using EFA, Rasch analysis, and hypothesis testing with a sample of migrant workers in South Korea. Our study indicated that the HLS-MW exhibited good reliability and valid psychometric properties.

We found that more than one-third of the migrant workers had limited Korean language proficiency. This finding is consistent with previous research and a recent national survey reporting that 44%‐52% of migrant workers have limited Korean proficiency [17,47]. Insufficient Korean language proficiency leads to difficulty in understanding and obtaining health-related information, using medical services, and communicating with health providers [23]. We discovered that only 34.1% (114/334) to 56% (187/334) of the survey respondents understood basic health-related words in Korean, such as “blood pressure” (item 2) and “temperature” (item 3),” and along with “sleep” (item 19) was the most difficult for workers to grasp in prose literacy.

The national Korean learning program for migrant workers primarily focuses on work-related issues (eg, understanding the Labor Standards Act and the Industrial Safety and Health Act). For this reason, migrant workers often encounter barriers to understanding and communicating with health care providers even after they pass EPS-TOPIK [20]. A recent analysis of Korean language textbooks for immigrants [48] also revealed that health-related topics were mainly confined to 1‐2 units of brief conversations at hospitals and pharmacies for the beginner level. Indeed, it is critical to include educational content addressing medical terminologies and health service use within the Korean language curriculum for migrant workers. In particular, migrant workers commonly experience musculoskeletal problems and allergies due to their working conditions and sleep disturbance in relation to working hours and shifts [49,50]. The retained items of the HLS-MW were selected to focus on situations encountered at the reception when visiting a doctor’s clinic for treatment or outpatient appointments and clarifying a doctor’s prescription for health problems. Additionally, questions were posed regarding situations in which medication is prescribed. These represent typical situations migrant workers living in Korea experience when facing health problems. Screening immigrants with low health literacy using short-form tools could be useful for health care providers who are unfamiliar with other ethnic populations, and could also serve as a resource for migrant workers to inform them of their health care weaknesses and allow them to receive support. In the future, it is necessary to develop educational content for Korean language and medical services in textbooks and educational programs for migrant workers, tailored to their level of Korean proficiency.

Women and those with a longer residence, younger age, higher education, and greater language proficiency have been associated with higher health literacy among migrant workers [29,51-54]. While longer residency in South Korea and Korean language proficiency were significantly associated with HLS-MW scores in this study, sex, age, and education were not. These results may be a simple artifact of sampling bias. For example, most participants in the study were men (82.9% or 277/334) and youths in their 20s and 30s. It is unclear why education was not associated with health literacy in the study. One possibility might be that migrant workers under the EPS in South Korea are primarily employed in small workplaces, doing similar jobs regardless of their education level; therefore, their exposure to Korean words may have been similarly limited, independent of their educational level. Further research is warranted to include, in the study sample, diverse migrant workers with more women and older workers.

There are certain study limitations to note. This validation study used a convenience sample of migrant workers; almost all (97% or 324/334) were documented workers. While there are no accurate statistics regarding the number of undocumented migrant workers in South Korea, one available government report estimates that there are nearly 390,000 illegal immigrants in South Korea, approximately 19% of all immigrants in the country [55]. Undocumented migrant workers are more likely to have frequent health problems and unmet health care needs compared to native workers [56]. Additionally, the proportion of H-2 migrant workers who responded to our survey with proficient Korean skills was 57.2% (191/334), compared to 4.2% (14/334) of E-9 migrant workers. H-2 visa holders (eg, Korean-Chinese) have higher Korean language proficiency than those with E-9 visas [10]. The sample also consisted of mostly male (277/334, 82.9%). Future validation of the HLS-MW should explore whether visa type or any other characteristics, such as documentation status and gender, are associated with health literacy among migrant workers.

Additionally, the HLS-MW does not suggest a cutoff point for detecting limited health literacy; therefore, more research is needed to develop a cutoff point. Since there exists no gold standard that determines who belongs to the low versus high health-literacy group in South Korea, research identifying a criterion for health literacy in Korean health care settings may be useful. Lastly, we conducted this study exclusively on the web. Considering that this study was conducted during the COVID-19 pandemic and that nearly 90% (288/331) of migrant workers in the sample had graduated from high school or higher, had an average age in the 30s, and depended on diverse information from EPS apps and web-based services, the study’s participants primarily responded using their mobile phones. Future research is necessary to increase the validity and dimensionality of the scale using an offline survey. Health literacy for migrant workers has not been studied in South Korea partly due to the lack of a scale to adequately measure their health literacy. We hope this scale will encourage researchers to explore the significance of problems regarding low health literacy in the health care context and enable health care providers to develop strategies to enhance migrant workers’ literacy skills.

### Conclusions

Despite the limitations, the HLS-MW is a valid and reliable scale for measuring the health literacy of migrant workers in South Korea. In particular, our findings shed light on the health experience among unskilled migrant workers from South Asia. Additional studies are warranted to further test the psychometric properties of the HLS-MW among diverse groups of migrant workers in South Korea while also establishing cutoff points to help identify those requiring health literacy support.

## Supplementary material

10.2196/59293 Multimedia Appendix 1 Correct rate of respondents (N=344).

10.2196/59293 Multimedia Appendix 2 Scree plot of the eigenvalues of the health literacy scale for unskilled migrant workers.

10.2196/59293 Multimedia Appendix 3 Comparison of health literacy scores and participants’ ability (theta).

10.2196/59293 Multimedia Appendix 4 Test information curve of the health literacy scale for unskilled migrant workers.

## Abbreviations

CFI: comparative fit index
CTT: classical test theory
CVI: content validity index
EFA: exploratory factor analysis
EPS: Employment Permit System
EPS-TOPIK: Employment Permit System—Test of Proficiency in Korean
HLS-MW: health literacy scale for unskilled migrant workers
ICC: intraclass correlation coefficient
IRT: item response theory
MNSQ: mean-square fit statistics
RMSEA: root mean square error of approximation
SRMR: standardized root mean square residual
TLI: Tucker-Lewis index
WHO: World Health Organization
WLSMV: weighted least square mean and variance adjusted
ZSTD: standardized fit statistics
