# Supplementary material for: Developing and Validating the Health Literacy Scale for Migrant Workers: Instrument Development and Validation Study
Source: JMIR Public Health Surveill. 2024 Nov 13;10:e59293. doi: 10.2196/59293 (PMC11577969; doi:10.2196/59293)
Supplement: Multimedia Appendix 1 [file publichealth-v10-e59293-s001.docx]

**Multimedia Appendix 1.** Correct rate of respondents (N=344).

|  | Response | Number of correct persons | Correct rate (%) |
| --- | --- | --- | --- |
|  |  |  |  |
| 1 | At the reception desk- Check in | 174 | 52.1 |
| 2 | Blood pressure | 113 | 33.8 |
| 3 | Temperature | 188 | 56.3 |
| 4 | At the doctors’ office 1 - Cough | 188 | 56.3 |
| 5 | Breathe | 158 | 47.3 |
| 6 | Antibiotics | 171 | 51.2 |
| 7 | Symptoms | 185 | 55.4 |
| 8 | At the doctors’ office 2- Ointment | 209 | 62.6 |
| 9 | Painkiller | 160 | 47.9 |
| 10 | Physical therapy | 171 | 51.2 |
| 11 | At the doctors’ office 3 - Allergy | 153 | 45.8 |
| 12 | Pain relief patches | 161 | 48.2 |
| 13 | Water | 184 | 55.1 |
| 14 | Assessment instruction 1- Blood sampling room | 151 | 45.2 |
| 15 | Prescription | 182 | 54.5 |
| 16 | Assessment instruction-2-Fast | 194 | 58.1 |
| 17 | Chaperon (guardian) | 150 | 69.5 |
| 18 | Outpatient | 117 | 35.0 |
| 19 | Health management 1- Sleep | 111 | 33.2 |
| 20 | Health management 2 – Health examination | 233 | 69.8 |
| 21 | Smoking | 184 | 65.6 |
| 22 | Appointment instruction (day) | 202 | 60.5 |
| 23 | Appointment instruction (place) | 201 | 60.2 |
| 24 | Appointment instruction (time) | 41 | 12.3 |
| 25 | Outpatient schedule (Department) | 209 | 62.6 |
| 26 | Outpatient schedule (day) | 189 | 56.6 |
| 27 | Nutrition Facts | 158 | 47.3 |
| 28 | Sodium | 50 | 15.0 |
