# Supplementary material for: Developing and Validating the Health Literacy Scale for Migrant Workers: Instrument Development and Validation Study
Source: JMIR Public Health Surveill. 2024 Nov 13;10:e59293. doi: 10.2196/59293 (PMC11577969; doi:10.2196/59293)
Supplement: Multimedia Appendix 2 [file publichealth-v10-e59293-s002.docx]

**Multimedia Appendix 2.** Scree plot of the eigenvalues of the HLS-MW.
