# Supplementary material for: Developing and Validating the Health Literacy Scale for Migrant Workers: Instrument Development and Validation Study
Source: JMIR Public Health Surveill. 2024 Nov 13;10:e59293. doi: 10.2196/59293 (PMC11577969; doi:10.2196/59293)
Supplement: Multimedia Appendix 3 [file publichealth-v10-e59293-s003.docx]

**Multimedia Appendix 3.** Comparison of health literacy scores and participant’s ability (theta).

| Health literacy score | Theta | Std. error |
| --- | --- | --- |
| 0 | -3.99 | 1.86 |
| 1 | -2.70 | 1.06 |
| 2 | -1.89 | 0.79 |
| 3 | -1.35 | 0.69 |
| 4 | -0.92 | 0.63 |
| 5 | -0.54 | 0.60 |
| 6 | -0.18 | 0.59 |
| 7 | 0.16 | 0.59 |
| 8 | 0.52 | 0.61 |
| 9 | 0.91 | 0.64 |
| 10 | 1.35 | 0.69 |
| 11 | 1.90 | 0.80 |
| 12 | 2.73 | 1.07 |
| 13 | 4.03 | 1.86 |
