# Supplementary material for: Developing and Validating the Health Literacy Scale for Migrant Workers: Instrument Development and Validation Study
Source: JMIR Public Health Surveill. 2024 Nov 13;10:e59293. doi: 10.2196/59293 (PMC11577969; doi:10.2196/59293)

**Multimedia Appendix 4**. Test information curve of the health literacy scale for unskilled migrant workers.

The horizontal axis is the participant’s ability, and the vertical axis is the true score. The true score ranges from o to the number of items (13 items). Test characteristic curve provides a means of transforming ability scores to true scores. The test information is calculated based on the summation of all the item information.


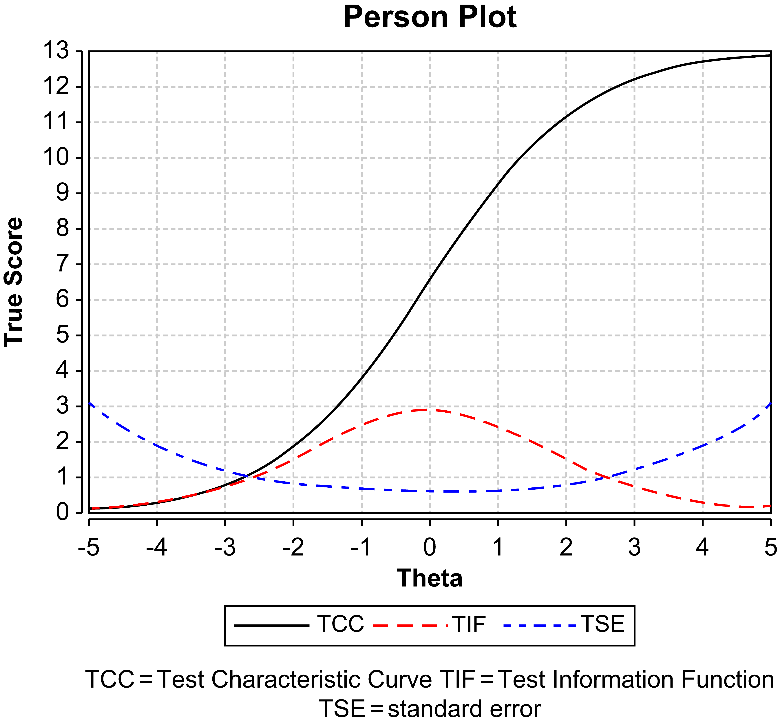

Supplement: Multimedia Appendix 4 [file publichealth-v10-e59293-s004.docx]
